# A Bionic Camera-Based Polarization Navigation Sensor

**DOI:** 10.3390/s140713006

**Published:** 2014-07-21

**Authors:** Daobin Wang, Huawei Liang, Hui Zhu, Shuai Zhang

**Affiliations:** 1 University of Science and Technology of China, Hefei 230027, China; E-Mail: zsustc@mail.ustc.edu.cn; 2 Institute of Advanced Manufacturing Technology, Chinese Academy of Sciences, Changzhou 213000, China; E-Mails: hwliang@iim.ac.cn (H.L.); hzhu@iim.ac.cn (H.Z.)

**Keywords:** bionic navigation, polarization measurement, camera based, *Cataglyphis*

## Abstract

Navigation and positioning technology is closely related to our routine life activities, from travel to aerospace. Recently it has been found that *Cataglyphis* (a kind of desert ant) is able to detect the polarization direction of skylight and navigate according to this information. This paper presents a real-time bionic camera-based polarization navigation sensor. This sensor has two work modes: one is a single-point measurement mode and the other is a multi-point measurement mode. An indoor calibration experiment of the sensor has been done under a beam of standard polarized light. The experiment results show that after noise reduction the accuracy of the sensor can reach up to 0.3256°. It is also compared with GPS and INS (Inertial Navigation System) in the single-point measurement mode through an outdoor experiment. Through time compensation and location compensation, the sensor can be a useful alternative to GPS and INS. In addition, the sensor also can measure the polarization distribution pattern when it works in multi-point measurement mode.

## 1. Introduction

Navigation technology plays an important role in human activities [1]. Up to now, a lot of navigation systems have been developed [2–5], such as INS, radio navigation systems, satellite navigation systems, celestial navigation systems and so on. An INS is a navigation aid that uses a computer, motion sensors (accelerometers) and rotation sensors (gyroscopes) to continuously calculate the position, orientation, and velocity of a moving object via dead reckoning without the need of external references. However the errors will accumulate with time so that the output data cannot be used after a long period of time. Radio navigation systems use radio signals for navigation, but they are susceptible to interference from external electromagnetic signals in the environment, so their accuracy is very low. As a typical satellite navigation system, GPS has been widely used in the world due to its ability to work in all weather conditions and its high precision. However, the GPS signal is usually very weak and becomes unreliable or even invalid in certain environments, such as tunnels, waterbodies and woods.

In addition, the GPS signal is easily shielded and interfered with in some special environments like military applications. Celestial navigation, also known as astronavigation, is an ancient art and science of position fixing that enables a navigator to transition through a space without having to rely on estimated calculations, or dead reckoning, to know their position. Celestial navigation uses “sights” or angular measurements taken between a celestial body (the sun, the moon, a planet or a star) and the visible horizon. It has a high precision and no cumulative errors, but the necessary equipment is usually very expensive and bulky. Although all of these navigation methods have been used widely in the world, each one of them has its disadvantages, and is not suitable as a sole means of navigation and positioning. Some places (for example, Mars, deserts, and at sea) have few things can be used for navigation and positioning, particularly Mars that even has no usable magnetic field, but in these places, the sunlight is still available, which is a good option for direction finding. According to Fox and his colleagues, polarized light caused by Rayleigh scattering in the Martian atmosphere is sufficient to be readily measurable [6]. Thus more and more researchers have begun looking for a new, cost effective, anti-jamming and all-weather conditions navigation and positioning method.

Recent biological research found that some animals can utilize the polarization direction of skylight to guide their navigation behaviors. In 1949 Von Frisch found that bees can make use of ultraviolet polarized light for navigation, which was the earliest public report on the polarization navigation capability [7]. Muller and Wehner found that *Cataglyphis* can use their compound eyes to obtain the polarization direction of skylight. Their eyes are very similar to a compass [8,9]. Dacke found that the wolf spider can perceive ultraviolet polarized light for three-dimensional navigation, and the research team also found that African dung beetles can use weak polarized moonlight for navigation to make themselves walk along a straight line [10]. Reppert *et al.* (2004) found that the North American monarch butterflies can use the sun as an auxiliary compass with ultraviolet polarized light for navigation during their migration process [11]. Homberg and Heinze found that a region in the brain nerves of grasshoppers and crickets is responsible for the polarization light navigation [12]. All of these researches in the biological field have laid a solid foundation for the design and development of a polarization sensor.

Generally speaking, the development of polarization measurement methods could be classified into two categories: point-source based and image based. Since the point-source based approach can only get polarization information of a point, this method is not robust enough and is vulnerable to be interference by external sources. For example: Lambrinos *et al.* equipped a robot with a point-source polarization sensor for its navigation [13]. This was an inchoate design of the polarization sensor. The sensor was composed of six photodiodes and polarizers. It can only measure one point in a time and the error of the sensor was fairly large. Later Chu developed a polarization navigation sensor based on the point-source principle [14,15]. Although the sensor was verified to be more accurate than the optical encoder according to his papers, it was vulnerable to environmental interference (such as clouds, masks, *etc*.) because it can measure only a single point in the sky. It also had six photodiodes and polarizers. Fan realized autonomous navigation with a bionic polarized sensor [16]. This sensor also consisted of photodiodes. Image-based polarization sensors also appeared very early. The image-based approach can obtain the polarization information of a region, but the light intensities of three different polarization directions are very difficult to collect accurately for the image-based sensor since there are too many difficulties to overcome, such as using motors, multi-camera data processing and so on. For example: Horvath *et al.* developed a three-lens, three-camera, full-sky image-based polarization meter [17]. They arranged the three cameras in a straight line, and the lenses they used were fisheye lenses. The fisheye lens could help them to obtain the all-sky polarization distribution, but meanwhile, it also could produce severe distortions. In addition, the sensor was not an automatic device. Sarita *et al.* developed a real-time, autonomous, polarization sensor which had been used on Mars [18]. Chahl *et al.* presented a new way to calculate the polarization direction [19] and successfully applied it on an image sensor. Sarkar proposed an image-based polarization meter [20]. The polarizer of the sensor needed to be manually rotated, so it was difficult to guarantee the consistency of the rotation angle, and it cannot collect data in real-time. In addition, it was a CMOS-based sensor. Currently, more and more image-based polarization meters use an external motor to rotate the polarizer, which can guarantee the consistency of the rotation angle, but the real-time performance is still poor because the response time of the motor is generally hundreds of milliseconds. The development of the polarization sensor is the foundation of the research on the navigation method based on polarized light. However, research on navigation methods based on polarized light is also necessary. Considerable works have been done in this field. For example: due to the fact that polarized light is widely distributed in the world, Lerner proposed a navigation method which works according to the polarization distribution in clear and turbid waters [21]. Yan established a high-precision polarization navigation model to verify the bionic navigation mechanism. He developed an optoelectronic measurement system, which can measure the angle between the carrier heading and the solar meridian [22]. Although fruitful results have been achieved, there are still a lot of problems about detection of polarization information that need to be solved.

In this paper, a real-time three-channel camera-based polarized light sensor is developed. The developed sensor has three cameras, and the three cameras are arranged in a triangular layout. The sensor has both the advantages of both the point-source based sensor and the image-based sensor. It can measure the polarization information of one point and also can measure the polarization pattern of a region. It has many advantages. Firstly, it obtains the three-channel signal synchronously through three cameras. Its frame rate can reach up to 20 fps, in other words, we can get a measurement data within 50 ms. Secondly, because every datapoint used is obtained from numerous CCD pixels by filtering, the sensor is very robust and less susceptible to interference by the external environment, especially in the single-point measurement mode. The maximum measurement error of the sensor is 0.3256° in the single-point measurement mode according to the indoor calibration experiments.

This paper is organized as follows: the work principle and the structure of the sensor are described in Section 2. The sensor, GPS and INS are compared in Section 3 through several experiments, and the experimental results in the multi-point measurement mode are also introduced. Some conclusions which can be drawn from this paper are given in Section 4, in addition, some future work is also discussed in this section.

## 2. The Work Principle and the Structure of the Sensor

### 2.1. The Measurement Principle of the Polarized Light

**Figure 1.**
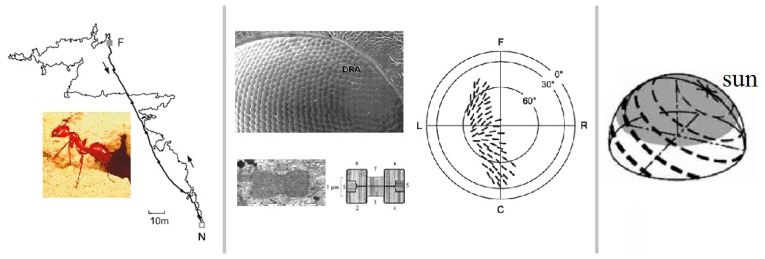
**Left:** The path integration of *Cataglyphis* for foraging and returning to the nest. *Cataglyphis* can navigate and positioning according to the polarization direction of the skylight and the odometer on their own. **Middle:** It includes the micrograph of the *Cataglyphis*' eye, the rhabdom of dumb-bell shape and its structure diagram. The rhabdom comprises two substances, which are mutually perpendicular. Such configuration enables the *Cataglyphis* to obtain the strength of the polarized light on one direction. In addition, it also includes the zenith projection of the array of the rhabdoms of the right eye of *Cataglyphis bicolor*. The black bars indicate the e-vector tuning axes of the (contralaterally looking) polarization analyzers. 0° horizon, C caudal, F frontal, L and R left and right visual hemisphere. **Right:** Three-dimensional representation of the polarization pattern under clear sky for 60° elevation of the sun. The bars indicate e-vector orientations as observed from the center of the hemisphere. Width of the bars represents the polarization degree.

*Cataglyphis* ants can travel thousands times of their body length to their nests in the Sahara desert. How can they accomplish this navigational task? Wehner has done many experiments on *Cataglyphis* and thought that the polarization sensitive organ within the compound eyes can perceive the polarization information of the skylight. The polarization pattern of skylight is relatively stable under clear-sky conditions, therefore, *Cataglyphis* ants can obtain their heading through the polarization information and complete the navigational task. Figure 1 shows the homing ability of the *Cataglyphis*, the structure of the rhabdom, the array form of the rhabdoms and the pattern of polarization under clear sky. The rhabdoms are the sensitive organs within their compound eyes. As shown in the figure, the rhabdom comprises two substances, which are mutually perpendicular. Such a configuration is the reason why the *Cataglyphis* can get the strength of the polarized light on one direction. Inspired by this, some researchers use a polarizer covering a camera to obtain the intensity of the polarized skylight on one direction through the picture collected by the camera. In addition, some interesting rules of the distribution of the rhabdoms' direction could be found by observing the array form of the rhabdoms. For example, most of them are concentrated in the directions of 0°, 30°, 45°, 90°, 120°, 135° and so on. The rhabdoms are extremely sensitive to the polarized light of these directions. The phenomenon is in line with the principle of the polarized light measurement, because the polarization degree and the polarization angle can be calculated more easily according to the intensity of the polarized light on these directions. In this paper, three directions are chosen, which are 0°, 45° and 90°, respectively.

The polarization information is generally represented with the Stokes vector S = (I, Q, U, V). In which, I, Q, U represent the total light intensity and the two orthogonal directions light intensity, respectively. The polarization angle could be determined according to Q and U, which are the characterization parameters to describe the polarity direction of the polarized light. And V represents the intensity of the circularly polarized light. Generally speaking, V is negligible. So the polarization information of the polarized light (such as the polarization degree and the polarization angle) can be calculated according to I, Q and U. The polarization measurement principle is that: the polarized light intensity of one direction is the function of the direction, which can be expressed by Equation (1):

(1) $$I^{\prime }(\phi )=\frac{1}{2}\left(I+Q\cos(2\phi )+U\sin(2\phi )\right)$$

where, I′(ϕ) is the polarized light intensity of the direction ϕ. According to Equation (1), there are three unknown variables, so it's necessary to measure the polarized light intensities of three different directions. As described above, we chose 0°, 45° and 90°. Then we can solve I, Q and U according to the light intensity of the three directions. After solved I, Q, U, we can calculate the corresponding polarization degree and polarization angle by using Equation (2). P and χ in Equation (2) are the polarization degree and polarization angle, respectively. The polarization degree indicates the proportion of the polarized light intensity in the total light intensity, and the polarization angle indicates the polarity direction of the polarized light:

(2) $$\{\begin{array}{c} P=\frac{\sqrt{Q^{2}+U^{2}}}{I}\\ \chi =\frac{1}{2}\arctan(\frac{U}{Q}) \end{array}$$

### 2.2. The Structure of the Sensor

**Figure 2.**
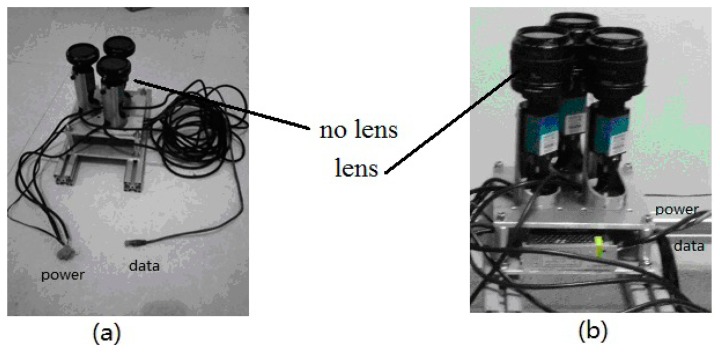
The polarization navigation sensor.

The sensor consists of three modules: the polarized light detection module, the power module and the data communication module, as shown in Figure 2. The power module includes a transformer and a regulator. The data communication module includes a Gigabit Ethernet switch and a few network cables. The polarized light detection module includes three cameras and three linear polarizers. When the lenses are not installed, the sensor works in the single-point measurement mode, as shown in Figure 2a. When the lenses (focal length, 28 mm) are installed, the sensor works in the multi-point measurement mode, as shown in Figure 2b. Of course, under some weather conditions the sensor needs to be installed a blue filter on the top of the three polarizers to make the output of the sensor more stable and accurate.

**Figure 3.**
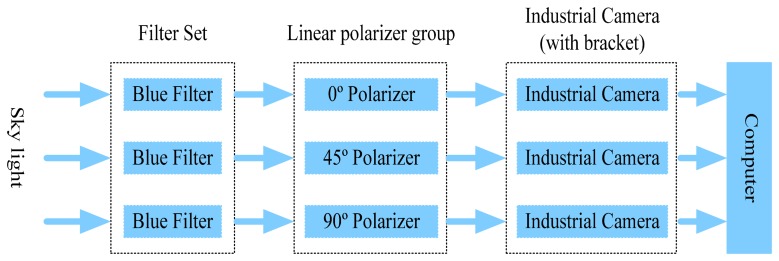
The configuration of the sensor.

To simplify the calculation process, the polarity directions of the three polarizers are installed at 0°, 45° and 90° with respect to the reference direction of the sensor, respectively. The block diagram of the navigation sensor is shown in Figure 3. The sky light passes through the blue filters and the three polarizers and into the industrial cameras. Then the industrial cameras collect all the polarized pictures into the computer for processing.

**Figure 4.**
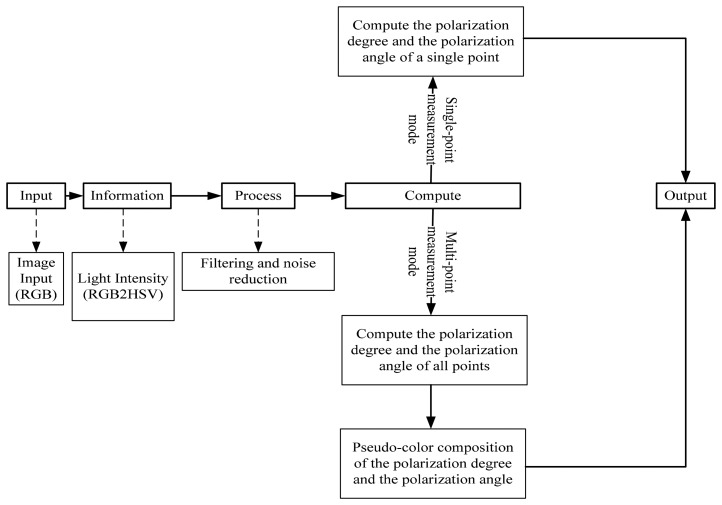
Data processing procedure.

The calculation process of the polarization information (the polarization degree and the polarization angle) is shown in Figure 4. The computer collects the image data of the three cameras and gets the polarized light intensities of the three directions. Then the computer calculates the polarization degree and the polarization angle according to the above equations. Finally, the computer output the final results.

### 2.3. The Single-Point Measurement Mode

In the single-point measurement mode, the sensor is a point-source based polarization measurement instrument. It can measure the polarization degree and the polarization angle of the zenith quickly. Real-time data collection and good robustness are required in this operation mode, so in this mode five steps are taken to improve the performance of the sensor.

#### Preprocessing

The cameras themselves usually produce some system noise, which is impacted by the ambient temperature, humidity of the environment and the consistency of the camera. For example, salt-and-pepper noise can usually be seen on images which from CCD cameras. This noise presents itself as sparsely occurring white and black pixels. An effective noise reduction method for this type of noise is a median filter. Increasing the exposure time of the camera is very similar to the median filter. Therefore we increase the exposure time of the camera as long as possible to make sure the output images of the camera clear and stable. However, excessive exposure time will produce camera over-exposure. This is the preprocessing step to the images from the cameras.

#### Cut and Down-Sampling

The three cameras are DFK23G445 from Imaging Source Company (Bremen, Germany). The resolution of this camera is 1280 × 960. Large datasets have a bad effect on the real-time data processing and the data from the edge of the image is less important than the data from the center, so we cut a picture with the size of 128 × 96 from the image's center. However, there are still more than one thousand pixels in each picture and so much data is still not easily to handle, so we use the down-sampling technique to reduce the picture to a size of 12 × 9. The down-sampling is to zoom out the image through the linear interpolation or the bilinear interpolation. The method used in this paper is bilinear interpolation.

#### Filtering

By the frequency domain analysis of the values of the 108 pixels, the probability distribution of each value could be obtained. The value with a probability less than a threshold (10%) is then removed and the expectation and standard deviation of the residual data is calculated. The expectation will be the output result of the camera if the standard deviation is less than 0.2, else increase the threshold and try again. This step is similar to a Gaussian filter. At the end we calculate the expectation of the remaining data as the output of the camera. Through filtering we can remove the Gaussian noise of the camera and also can effectively eliminate the problem of occlusion. This is a major advantage of this sensor. For example, the sensor is still able to obtain a stable and accurate output even if there is a bird or an airplane flying over the sensor, but most current point-source devices cannot do that because they use the POL and the photodiode as its sensitive components.

#### Separate the acquisition and processing of the data

In order to obtain a faster processing speed many things have been done to optimize the program. Data acquisition and data processing are written into two separate threads respectively. This can greatly improve the computing efficiency. The display operations of the images are reduced and OpenCV is used to process the pictures. These things could assurance the procedures run efficiently and quickly.

#### Calibration

Because the polarity direction of the three polarizers cannot be installed at the true values of 0°, 45° and 90°, the sensor usually has some nonlinear noises. Since there are some slight differences among the three cameras, it will also produce some noises. In order to reduce these noises, a Fourier function is used to fit in with the characteristic curve of the sensor as follows:

(3) $$\begin{array}{cc} f(x)=a_{0}+a_{1}\cos(\omega x)+b_{1}\sin(\omega x)+\cdots +a_{n}\cos(n\omega x)+b_{n}\sin(n\omega x) & x\in D_{i} \end{array}$$

where D_i_ is a range of the output value of the sensor and D_i_ ∈ [−90°,90°]. The a_0_, a_1_, b_1_, a_n,_ b_n_ and ω are the parameters of the characteristic curve of the sensor.

### 2.4. The Multi-Point Measurement Mode

In the multi-point measurement mode, the sensor can obtain the polarization pattern of a region in the sky. It can monitor the changes of the polarization pattern of skylight with time. It can also monitor the changes of the polarization distribution pattern under different weather conditions.

As described in Section 2.2, in this mode the hardware of the sensor needs some changes: three suitable camera lenses are necessary. The focal length of the lenses could be chosen according to the needs of the experiments. In this paper, three 28 mm Canon lenses have been chosen. This is because the sensor cannot only observe the detail polarization distribution information (the polarization degree and the polarization angle), but also have a suitable and reasonable wide field of view in this focal length.

In this mode, the sensor can obtain the polarization information of a region near the zenith of the sky within 200 ms and can continuously collect the polarization distribution of the sky. So the advantage of this mode is that the sensor can observe the dynamical changes of the polarization distribution of the sky.

### 2.5. The Coaxiality of Three Cameras

Since the mounting positions of the three cameras are different, the view fields of the three cameras are different, too. To achieve more accurate polarization information, the optical axes of the three cameras have to be arranged coaxially, and the planar orientations of the three cameras are also the same. Firstly, we try our best to machining the equipment parts with high precision. The precision of equipment parts are all ±10 μm. This can ensure that the three cameras have a good coaxiality. Then Zhang Zhengyou's calibration method [23] is adopted to adjust the planar orientations of the cameras through a checkerboard calibration pattern.

## 3. Experiment

### 3.1. Indoor Calibration

**Figure 5.**
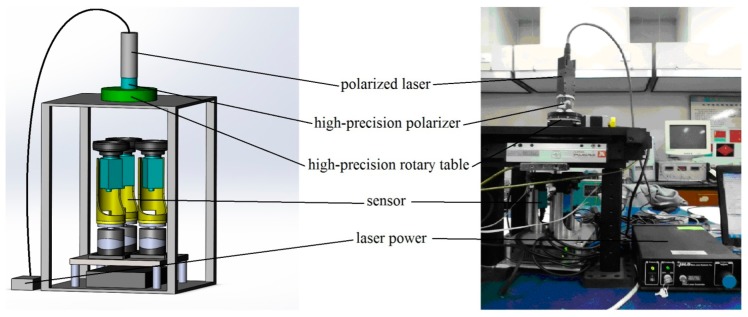
**Left:** diagrammatic sketch of the polarized light. **Right:** The polarized light.

The error of the polarity direction of the three polarizers will produce some nonlinear noises. In order to reduce the noises, an indoor calibration experiment of the sensor through a polarized light is done in this section. The polarized light and its diagrammatic sketch are shown in Figure 5. The polarized light is composed by a polarized laser, a laser power, a high-precision polarizer, a high-precision rotary table and so on. The error of the rotary table is 0.01°.

Firstly, the polarity directions of the cameras need to be calibrated. We rotate the rotary table and adjust the polarity direction of the polarized light to 90°, then rotate one polarizer and adjust the polarity direction of the polarizer to make the output of the camera to a minimum value. By this way, the polarity direction of the camera could be adjusted to 0°. Similar to that described above, we could adjust the polarity direction of the other two cameras to 45° and 90°, respectively.

**Figure 6.**
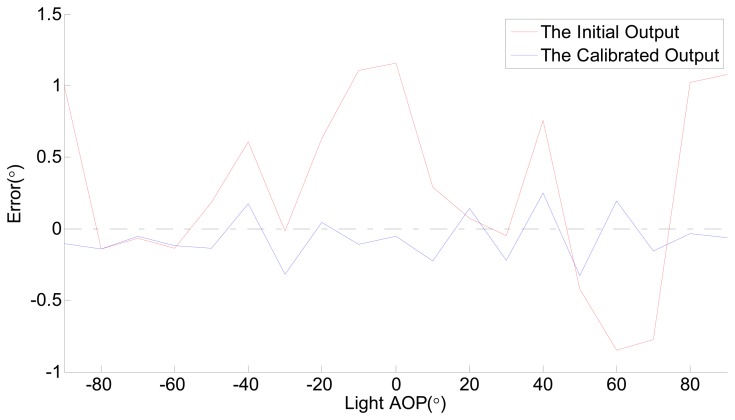
The error of the sensor before and after calibration.

Secondly, the nonlinear error of the sensor also needs to be reduced. We rotate the rotary table from −90° to 90° to rotate the polarity direction of the polarized light from −90° to 90°. In this process, the output data of the sensor are collected once every 10°. The output data of the sensor has been filtered as described in Section 2.3 (Step 3). The error of the initial data in the experiment is shown in Figure 6. The most striking observation from Figure 6 is that the effect of the filtering is outstanding. The max measurement error of the AOP (angle of polarization) is 1.1556°. The results indicate that there are some slight errors which are described in Section 2.3 (Step 5). For this reason it is difficult to ensure the accuracy of the initial output data from this sensor. In order to obtain a higher accuracy, the sensor needs to be calibrated. As described in Section 2.3 (Step 5), a Fourier formula is used to fit in with the characteristic curve of the sensor. According to the result of the unprocessed output data of the sensor and the data of the polarized light, the parameters of Equation (3) are shown in Table 1.

By calibration, the initial output of the sensor is converted to the new data. After calibration, the error of the sensor is shown in Figure 6, too.

The RMSE and the max error of the polarization angle output of the sensor are 0.6503° and 0.3256°, respectively. The experimental data before and after calibration are both plotted in Figure 6, with the angle (Light AOP) on the *x*-axis and the error (Sensor AOP- Light AOP) on the *y*-axis.

### 3.2. Outdoor Calibration

**Figure 7.**
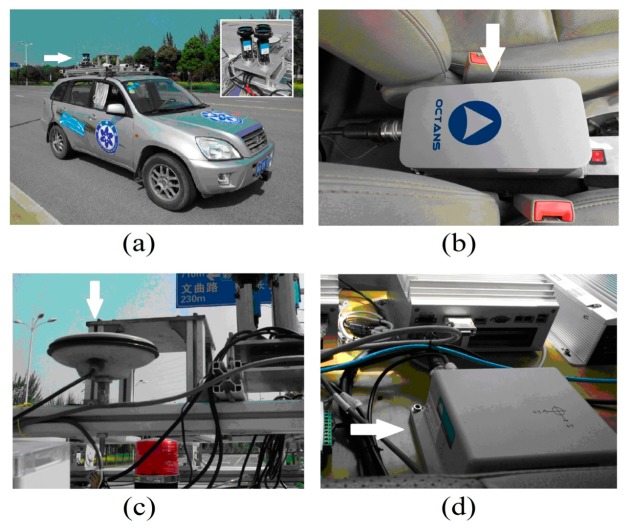
The experimental conditions.

The polarization sensor, GPS and INS were installed on one vehicle. The polarization sensor is mounted on the roof of the vehicle, as shown in Figure 7a. Figure 7b shows the Octans INS sensor made by IXSEA (city, France). It is mounted between the front seats. Its performance parameters are as follows:

Heading Accuracy: 0.1° × Secant Latitude
Heading Resolution: 0.01°
Heading Stabilization Time: <5 min

The GPS (SPAN-CPT), shown in Figure 7c,d, is made by NovAtel (Calgary, AB, Canada). It is a tightly coupled GPS+INS integrated navigation system, and includes two parts: the antenna and the data decoupling terminals. Its performance parameters are as follows:

Heading Dynamic Accuracy: 0.1° RMS
RTK Positioning Accuracy: 1 cm+1 ppm (RMS)
Gyro Zero Drift Stability: ±1°/hr

**Figure 8.**
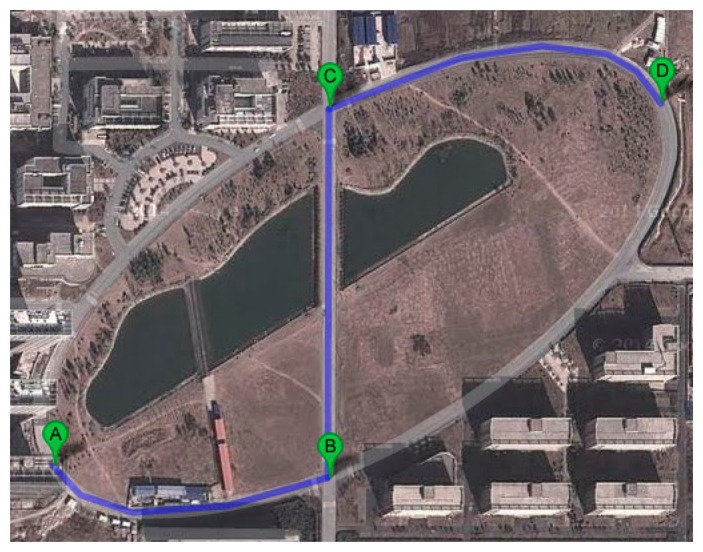
The ring road in the map.

Then the car is driven along a curved path and the computer simultaneously acquires and saves the data obtained from the three sensors. The path is shown in Figure 8.

**Figure 9.**
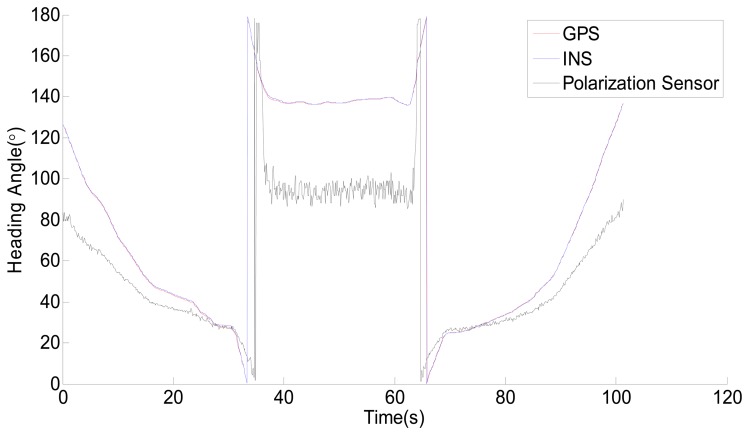
The raw data acquired from GPS, INS and the polarization sensor.

The raw data acquired from the three sensors is shown in Figure 9. The sampling period is 100 ms. In fact the sampling rate of the polarization sensor can reach up to 20 fps in the single-point measurement mode. In other words, an output data from the polarization sensor can be acquired in 50 ms. However, a sampling frequency of 10 fps is sufficient. The time and location of the measurements are always changing while the vehicle is moving, therefore, the measurement data of the sensor needs time and location compensation. According to the movement of the Sun, the polarization angle of the zenith will change as time goes on. Because of the movement of the vehicle, the position of the zenith also will change. The time compensation data can be obtained according to the time. The location compensation data can be obtained from the astronomical calendar according to the time and location. The output data shown in Figure 9 has been compensated with time. The process is simple. We increase 4.1667 × 10^−4^ degree per interval because the azimuth of the sun changes about 15° per hour. However, because the range of activity is limited and small, the influence of the movement of the vehicle could be negligible. In addition, because the output of the polarization sensor is in the range of 0° ∼ 180° and the output of GPS and INS are in the range of 0° ∼ 360°. To make it easier to see the relationship among GPS, INS and the polarization sensor, the output in the range of 180° ∼ 360° of GPS and INS is subdued 180°. From Figure 9, it is easy to see that the output of polarization sensor is significantly different with GPS and INS, however, there appears to be some relationship between them.

**Figure 10.**
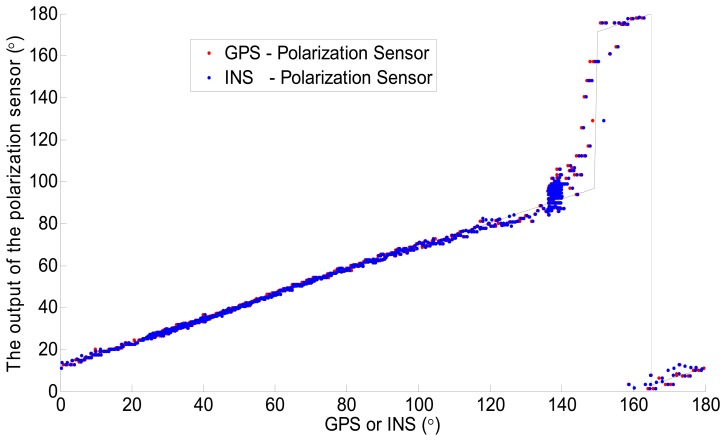
The characteristic curves of the polarization sensor with GPS and INS.

The characteristic curves of the polarization sensor with GPS and INS are plotted in Figure 10. As can be seen in the figure, the characteristic curves are both single-valued function. The two curves can be approximated by three straight line segments and the three straight line segments are not smoothly continuous. This phenomenon was caused by two reasons. One is the difference of the 0° reference direction among the polarization sensor, GPS and INS. The other is the non-linear error of the sensor which caused by the polarity direction errors of the three polarizers. The output of the polarization sensor can be easily corrected through a piecewise linear function. The formula is shown in Equation (4). However, the values of these parameters in Equation (4) are time dependent, so the sensor needs to be calibrated before use:

(4) $$f(x)=\{\begin{array}{cc} 0.5673x+12.2502 & 0\leq x<150\\ 0.5673x+86.3955 & 150\leq x<165\\ 0.5673x-90.6045 & 165\leq x\leq 180 \end{array}$$

**Figure 11.**
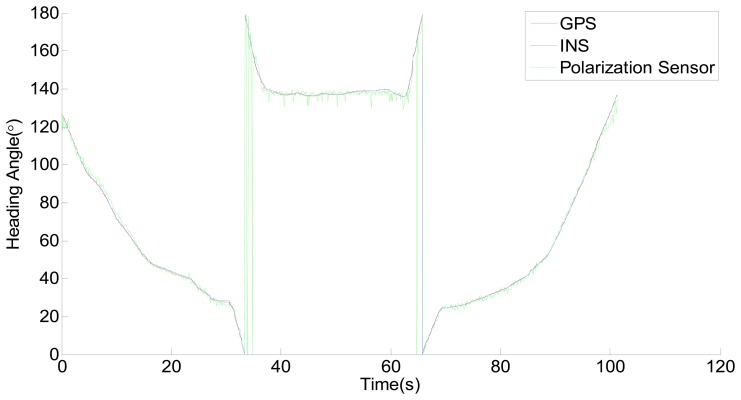
GPS, INS and the corrected result of the polarization sensor.

The raw data acquired from the polarization sensor are corrected to the new data according to the Equation (4) and the corrected result is shown in Figure 11.

**Figure 12.**
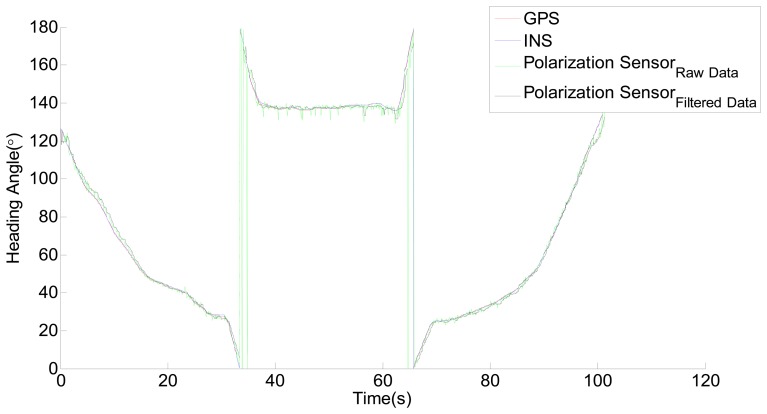
GPS, INS and the filtered result of the polarization sensor.

The roll and pitch changes of the moving vehicle can also have certain impact on the measurement of the polarization sensor. From the output data of the sensor, they could be easily categorized as high frequency noises, a low-pass filter is therefore utilized to solve the problem. The filtered result is shown in Figure 12.

### 3.3. Test in the Single-Point Measurement Mode

**Figure 13.**
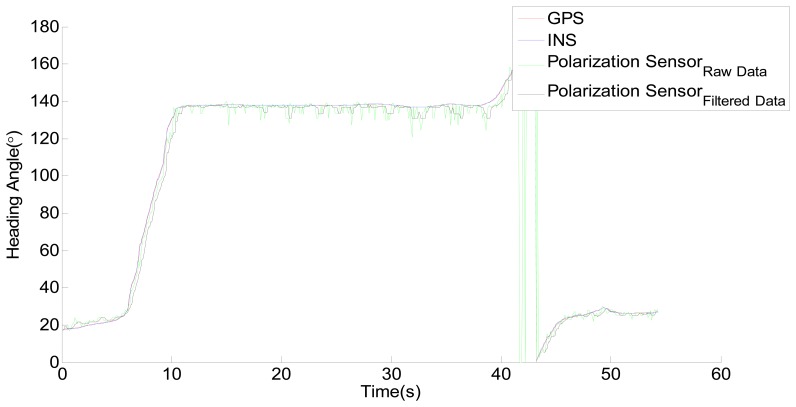
The result of GPS, INS and the polarization sensor.

In order to validate the feasibility of the correction method described above, another experiment with the sensor is performed later in the outdoor environment. The result of this experiment is shown in Figure 13. The clearest observation from the result is that the calibration method of the sensor proposed in this paper is feasible. In addition, the sensor can be used as a useful alternative to GPS and INS when it working in the single-point measurement mode.

### 3.4. Test in the Multi-point Measurement Mode

**Figure 14.**
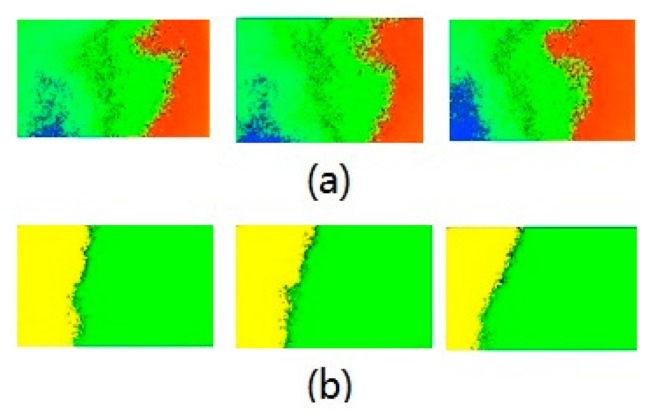
(**a**) The distribution of polarization degree in different time of a day; (**b**) The distribution of polarization angle in different time of a day.

In the multi-point measurement mode, every pixel needs to be corrected by the same method described above. Since there are more data that need to be processed, the sampling rate only can reach 1 ∼5 fps. In multi-point measurement mode, another experiment of the sensor in the outdoor environment is performed. Under clear sky conditions, the sensor is used to get the skylight polarization distribution pattern. Figure 14 shows the output of the sensor in different times of a day, in which Figure a is the distribution of polarization degree, and Figure b is the distribution of polarization angle. Every color in these pseudo-color composite pictures is a value of the polarization information. Under clear sky conditions, according to the Rayleigh scattering model, the polarization degree is distributed in a banded pattern, and the polarization angle has an axially symmetric distribution. It is easy to know that the polarization distribution obtained through the sensor is in line with the theoretical distribution model from Figure 14. The interval time of the three pictures in one group was about 15 min. It is obvious that the polarization distribution pattern is changing as time goes on, but the change is not remarkable, and can be compensated with the external information, for example, time, location, *etc*.

## 4. Conclusions and Future Work

The paper presents a real-time bionic camera-based polarization navigation sensor and introduces its working principle and structure. The properties of the sensor are also discussed. The sensor's frame rate can reach up to 20 fps and its accuracy can be maintained within 0.3256 degrees in the single-point measurement mode. In addition, it also can be seen as an image-based sensor and can measure the polarization distribution pattern of a region when it is working in the multi-point measurement mode. In this paper, the outdoor experiments with the polarization sensor have been done on a vehicle, and the performance of the sensor is compared with GPS and INS. A calibration method of the sensor for the outdoor experiments also is proposed; in addition, when the sensor works in the single-point measurement mode, it can effectively reduce or eliminate the effects of occlusion. The sensor proposed in this paper is highly effective, has a simple structure and low cost, and may play an important role in the navigation area in the future. However, due to the limitations of time and the length of the paper, there is still a lot of work that needs to be introduced. Firstly, the output range of the sensor in this paper is 0° ∼ 180° and needs to be expanded to 0° ∼ 360° for navigation experiments. Secondly, a detailed and feasible navigation and positioning method based on the polarization sensor needs to be proposed.

## Figures and Tables

**Table 1.** The parameters of Equation (3).

a_0_ = −457.3	a_4_ = −175
a_1_ = 830.6	b_4_ = −19.45
b_1_ = 121.3	a_5_ = 58.69
a_2_ = −620.3	b_5_ = 5.734
b_2_ = −67.97	a_6_ = −11.25
a_3_ = 373.3	b_6_ = −0.397
b_3_ = 41.07	ω = 0.01819
